# The Interrelation between Reactive Oxygen Species and Autophagy in Neurological Disorders

**DOI:** 10.1155/2017/8495160

**Published:** 2017-12-17

**Authors:** Congcong Fang, Lijuan Gu, Daniel Smerin, Shanping Mao, Xiaoxing Xiong

**Affiliations:** ^1^Department of Neurology, Renmin Hospital of Wuhan University, Wuhan, Hubei 430060, China; ^2^Central Laboratory, Renmin Hospital of Wuhan University, Wuhan, Hubei 430060, China; ^3^Department of Neurosurgery, Stanford University School of Medicine, Stanford, CA 94305-5117, USA

## Abstract

Neurological function deficits due to cerebral ischemia or neurodegenerative diseases such as Alzheimer's disease (AD) and Parkinson's disease (PD) have long been considered a thorny issue in clinical treatment. Recovery after neurologic impairment is fairly limited, which poses a major threat to health and quality of life. Accumulating evidences support that ROS and autophagy are both implicated in the onset and development of neurological disorders. Notably, oxidative stress triggered by excess of ROS not only puts the brain in a vulnerable state but also enhances the virulence of other pathogenic factors, just like mitochondrial dysfunction, which is described as the culprit of nerve cell damage. Nevertheless, autophagy is proposed as a subtle cellular defense mode against destructive stimulus by timely removal of damaged and cytotoxic substance. Emerging evidence suggests that the interplay of ROS and autophagy may establish a determinant role in the modulation of neuronal homeostasis. However, the underlying regulatory mechanisms are still largely unexplored. This review sets out to afford an overview of the crosstalk between ROS and autophagy and discusses relevant molecular mechanisms in cerebral ischemia, AD, and PD, so as to provide new insights into promising therapeutic targets for the abovementioned neurological conditions.

## 1. Introduction

Reactive oxygen species (ROS), an umbrella term for a category of active oxygen-containing compounds generated from aerobic metabolism [1], encompasses superoxide anion (O_2_^−^), hydrogen peroxide (H_2_O_2_), and free radical (superoxide and hydroxyl radicals). Each of these compounds can damage biomacromolecules essential for various cellular processes [2], while simultaneously playing an indispensable role in the redox signaling cascade required for critically important biological events [3]. ROS are likely to cause oxidative stress when the oxidation of ROS outweighs the antioxidation [4], which is believed to damage cells. Compared with other organs, the brain has the most active oxidative metabolism, with a high demand for oxygen. The brain's active oxidative metabolism combined with its deactivation of detoxification systems and severe deficiency of antioxidants jointly upsets the redox balance, causing immeasurable oxidative brain tissue injury. This process is closely correlated with the occurrence and development of cerebral ischemia and neurodegenerative diseases [5].

Autophagy is a precisely regulated biological process characteristic of eukaryotic cells during which the superfluous and damaged structures of cells are eliminated via lysosome degradation to maintain normal cellular physiological functions [6] for the purpose of adapting to all kinds of adverse stimuli. Existing studies suggest that autophagy is widely engaged in neuronal fate determination in diverse neurological conditions [7–9]. However, excessive autophagy can promote a programmed cell death, known as autophagic cell death or type II programmed cell death [10], which is morphologically distinct from apoptosis and necrosis. That is to say, moderate or optimal activation of autophagy is desirable, and neither excessive nor insufficient autophagy completely lacks toxicity to neurons [11].

ROS are associated with cell damage [12] and have traditionally been thought to function solely as unfriendly molecules, despite exposure to ROS being unavoidable for cells in an aerobic environment [13]. However, an increasing number of researchers have found that ROS can participate in various physiological processes as a kind of signaling molecule, including the induction of autophagy that is considered to be an effective defense mechanism against cellular stress [14, 15]. More importantly, it is the mitochondrial ROS which is a master inducer of autophagy under conditions of nutrient starvation, ischemia, or hypoxia [16–18]. Conversely, activation of autophagy is a key part of the cellular response to oxidative stress because the process disposes defective components before further damage/aggregation occurs [19]. In summary, the interaction and the balance between ROS and autophagy can be a key part of regulating cellular homeostasis.

It is well established that both ROS and autophagy are strongly associated with neurological diseases, but clarifying the functional relationship of the two mechanisms seems to be difficult because of their dual role in many disease processes. This review is designed to state the role of ROS and autophagy in neurological disorders and their underlying molecular mechanisms so as to offer novel strategies for the treatment of nervous system diseases.

## 2. Reactive Oxygen Species (ROS)

### 2.1. Generation and Scavenging of ROS

It is now well documented that mitochondria are the main source of intracellular ROS; 90% of which are derived from the respiratory chain on the mitochondrial inner membrane. The generation of mitochondrial ROS is initiated by the formation of O_2_^−^ via the combination of electrons leaking from the mitochondrial respiratory chain complexes (mainly complexes I and III) and O_2_. Highly active O_2_^−^ can then be transformed into more stable H_2_O_2_ in the presence of superoxide dismutase (SOD). The quick conversion of H_2_O_2_ into H_2_O can be catalyzed by catalase (CAT) and glutathione peroxidase (GSH-Px) and serves as the source of OH^−^ as well [3, 20, 21].

Under normal circumstances, ROS emissions in mitochondria are rather low and render minimal damage because mitochondria have potent antioxidant defense systems that sufficiently scavenge unneeded ROS. Whereas, unbridled ROS ensue only if mitochondria are subjected to deleterious incidents while simultaneously experiencing a drop in transmembrane potential. There is a positive-feedback mechanism called “ROS-induced ROS release” (RIRR) that accounts for the interaction between ROS and mitochondria. During RIRR, a burst of mitochondrial ROS is evoked by ROS, reducing mitochondrial membrane potential (MMP) and causing a longer opening of mitochondrial permeability transition pores (mPTP) [22]. Generally, moderate activation of mPTP is required for healthy mitochondrial metabolism. Once mitochondria are attacked by an inappropriate release of ROS, mitochondrial membrane depolarization interferes with mitochondrial respiratory chain function and can create a vicious circle provoking further ROS accumulation [23].

**Figure 1:**
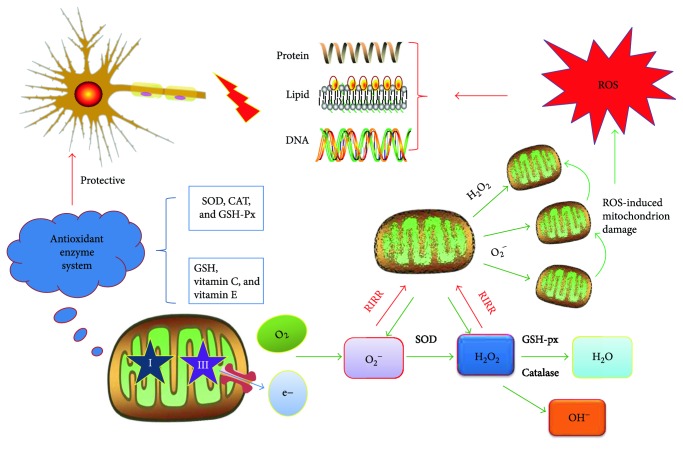
The generation and scavenging of reactive oxygen species (ROS) in mitochondria. The “→” refers to activation or induction, and the “⊢” refers to inhibition. Under normal or stress conditions, ROS is mainly born from the mitochondrial respiratory chain with the beginning of O_2_^−^ production, followed by the conversion to H_2_O_2_ then OH^−^ under the catalysis of SOD and GSH-px. Defective mitochondria can instigate ROS accumulation with a “RIRR” positive-feedback mechanism. Excessive ROS can inflict severe damage on biomacromolecules, which can be counteracted by the antioxidant enzyme system to some degree.

As mentioned, abnormally high levels of ROS can be quickly neutralized to cellular levels by a complex network of various robust antioxidants, which is essential for sustaining the normal functions of cells [24]. There are two major antioxidative systems that consist of enzymatic and nonenzymatic antioxidants. The former is represented by SOD, CAT, and GSH-Px, while the latter includes glutathione (GSH), vitamin C, vitamin E, and so forth. Nevertheless, when there exists any redox imbalance between the generation and the scavenging of ROS, oxidative stress occurs, leading to unpredictable oxidative damage to organelles, proteins, lipids, and DNA, as well as the disruption of cellular structures and functions and eventually cell death [25] (Figure 1).

### 2.2. Biomarkers of ROS/Oxidative Stress

Due to the nature of ROS, which are active for a relatively short lifespan, various complex and time-consuming detection means such as electron spin resonance and spin trapping technology are relatively difficult to practically implement, and the results are also often offset by the mixing of heterogeneous groups [26]. Therefore, ROS or oxidative stress level is usually measured by monitoring the activity of cellular antioxidant enzymes such as SOD and GSH-px, each of which can indirectly reflect the ability to remove ROS [27]. Concurrently, GSH and malondialdehyde (MDA) are used to mirror oxidative stress resistance and injury.

Superoxide dismutase, a copper-containing protein isolated from bovine red blood cells by Mann et al. for the first time in 1938, was rediscovered and named as SOD by Fridovich and Mccord in 1969. SOD is able to scavenge ROS. The glutathione peroxide (GSH-Px) is extensively present in the cytoplasm. Mitochondria contain two kinds of GSH-Px, GSH-Px1 and GSH-Px4, by which lipid peroxide induced by OH^−^ can be decomposed into the corresponding alcohol or peroxide-induced injury can be reduced [28]. As is widely known, common ROS involved in cellular damage are mainly OH^−^, H_2_O_2_, and O_2_^−^ [29]. The dynamic conversion among the three parts depends upon SOD and GSH-Px, only by which can O_2_^−^ be reduced into H_2_O, thus mitigating oxidation damage [30]. In conclusion, the activity of these two antioxidant enzymes may be the reflex of the ability to eliminate ROS [31] (Figure 1).

Glutathione (GSH) is the most abundant nonprotein thiol and broad-spectrum antioxidant in mitochondria and contains two forms: reduced glutathione (GSH) and oxidized glutathione (GSSG). The former accounts for about 95% of GSH and, as the primary ROS scavenger, can effectively remove H_2_O_2_ and O_2_^−^ and other free radicals, while concurrently being transformed into recyclable GSSG via glutathione reductase.

In the above processes, catalase (CAT) and glutathione reductase (GR) are typically used in combination with SOD, GSH-Px, and GSH as potential antioxidant biomarkers to evaluate oxidative stress.

There are also some other ROS measurement parameters based on oxidation of lipids, proteins, and DNA. Some examples of these parameters are MDA, 4-hydroxy-2-nonenal (4-HNE), 3-nitrotyrosine (3-NT), and 8-OHdG. Any accumulation of oxidation byproducts implies deterioration through oxidative damage, but different byproducts represent the different levels of cellular damage. Excess ROS inflict irreversible damage to nucleic acids, which has been reported to be an early event in oxidative damage. 8-Hydroxy-2′-deoxyguanosine (8-OHdG) is a biological index generated by the oxidation of DNA along with the loss of its integrity. As a pivotal biomarker of endogenous oxidative DNA, 8-OHdG levels have been commonly assessed to estimate ROS-induced DNA lesions in multiple neurological disorders.

As for malondialdehyde (MDA), the ubiquitous final product of lipid peroxidation created by ROS attacking unsaturated fatty acids of biological membranes [32], its accumulation can incur cross-linking polymerization of macromolecules such as protein and nucleic acid, permeability and destruction of membrane structures, and eventually cell death. The degree of lipid peroxidation can be estimated by the quantity of MDA in the tissue, so MDA is proposed to be one of the indicators of intracellular oxidative stress [33]. Similarly, 4-hydroxy-2-nonenal (4-HNE), a specific clinical detection index of polyunsaturated fatty acid peroxidation, is also currently utilized to measure the extent of oxidative lipid damage. Isoprostane is the best available index of lipid peroxidation because of its stability. In summary, these indicators may better assess oxidative damage of brain tissue because various polyunsaturated fatty acids are susceptible to ROS during oxidative metabolism in the brain.

Likewise 3-nitrotyrosine (3-NT), an important metabolite of oxidative lesions in protein, has been measured in brain tissue, with increased levels in PD and AD populations. 3-NT is stable both in vitro and in vivo. There is a lot of value in the assessment of oxidative stress for clinical research [34].

### 2.3. ROS/Oxidative Stress-Related Signal Pathway

Keap1/Nrf2/ARE cascades have proven to be the most important antioxidant defense, and almost all protective antioxidant genes contain antioxidant response elements (ARE). When exposed to oxidative stress, Keap1 (kelch-like ECH-associated protein 1) can be separated from Nrf2 (nuclear factor erythroid 2-related factor 2) by uncoupling activity or reduction of ubiquitination and degradation, then translocation of Nrf2 into the nucleus targeting ARE. Nuclear translocation leads to the expression of antioxidants and phase II detoxifying enzymes, which is shown to greatly reduce ROS and ensuing oxidative damage.

In addition, several intracellular signaling pathways related to redox state, such as PI3K/Akt, JNK, MAPK, and ERK, can dissociate Nrf2 from Keap1 through phosphorylation of Nrf2. These signaling pathways also cause Nrf2 to translocate to the nuclease and activate the antioxidant system, which is expected to augment the oxidative defense capacity [35, 36].

### 2.4. Reactive Oxygen Species (ROS) in Neurological Disorders

Loss of neurons is a key link in the pathophysiological process of nervous system diseases, which is mediated by oxidative stress, mitochondrial disturbances, abnormal protein aggregation, and so on [37]. One of the most important problems is oxidative stress, or ROS [38]. To our knowledge, the brain weighs just 2% of the body's weight, but its metabolic oxygen consumption accounts for 20% of total oxygen consumption of the organism under nonstress conditions. High oxygen demand is always accompanied by more ROS. The brain is rich in various polyunsaturated fatty acids sensitive to ROS, but is relatively devoid of antioxidant enzymes and GSH, adding that neurons are considered terminally differentiated cells [39], which make brain tissue more inclined to suffer damage from ROS [40, 41].

Robust evidence suggests that ROS display a recognized role in neuronal death after brain ischemia [42]. Either an initial burst of ROS induced by ATP consumption and mitochondria depolarization in the ischemic phase or the Ca+-dependent ROS generation at the reperfusion stage can pose a hazard for neurons [42]. As noted earlier, excessive production of ROS can not only damage cellular macromolecules but also impair antioxidant enzymes and nonenzymatic antioxidants during I/R insult, which is unfavorable for neurofunctional recovery. Sharma and Airao have shown that lipid oxidation byproducts such as MDA are markedly increased in ischemic tissues, but SOD, CAT, and GSH levels are reduced. Early administration of solasodine can ameliorate progressive ischemic injury through its potent antioxidant properties [43]. The Nrf2/ARE pathway is referred to as a potent defense mechanism against oxidative stress, which is expected to be a feasible direction of antioxidant treatment against ischemia-reperfusion (I/R) injury. In the Shah and Li study, they found that Nrf2 knockout mice in the I/R group present more obvious neurologic deficits than the wild type group with a significant increase in the area of infarction [44]. Enhancing the activation of Nrf2 by tBHQ, a natural Nrf2 inducer, can reduce and limit brain damage and is therefore possibly a practical prevention strategy for stroke-prone patients [45].

Studies have shown that the cellular damage in the early stages of AD is ascribed to oxidative stress [46], and notably, a large number of markers of oxidative stress are located in intracellular NFTs, a hallmark of the brains of AD patients [47]. A significant decrease in GPx and CAT activities and total GSH levels, which indicates a feeble antioxidant defense system in early AD, may facilitate the development of the disease. Meanwhile, extensive experiments collectively verify that antioxidants do delay the occurrence and progression of AD [48]. These oxidative stress indicators are used to characterize the earliest events of AD and are reliable tools for early diagnosis and prevention of AD [49].

Recent progress in PD has revealed that dopaminergic neurons are susceptible to oxidative stress because of inherent biological features. Clear evidences show that 4-HNE within such body fluids as CSF and serum is widely described as a clinical parameter of oxidative damage in PD individuals. Reactive (OH) and subsequent MDAs have been reported to be significantly increased in PD patients, which contribute to dopaminergic neuronal loss [50]. Nrf2 exists in the nigral dopaminergic neuron cytoplasm, but is located in the nucleus of age-matched PD patients, which strongly suggests that Nrf2 may contribute to combating oxidative brain damage via the transcription of genes encoding antioxidant enzymes [51]. Recent studies have claimed that upregulation of Nrf2 provides neuroprotection against oxidative stress-induced neurotoxicity in PD. Rb1 can enhance the transcriptional activation of Nrf2 and upregulate the expression of HO-1, an endogenous antioxidant enzyme and downstream effector of Nrf2, by modulating PI3K-mediated Nrf2-ARE signal pathway, which is shown to serve as a rational cytoprotective agent against oxidative insults of dopaminergic cells [52]. Taken together, ROS elevation initiates neuronal damage and we propose that Nrf2-related agents look set to offer an up-and-coming clinical therapy.

## 3. Autophagy

Autophagy was observed in mouse hepatocytes by Ashford and Porter for the first time in 1962 and visually described as cellular self-eating [53]. Nevertheless, it was De Duve that first came up with the concept of autophagy in 1967 [54]. Autophagy refers to macroautophagy in this review, the most common and well-studied form, which is distinguished from microautophagy and chaperone-mediated autophagy (CMA) by the different degradation pathways of substrates [55, 56]. Autophagy induction is a complicated and ordered multistep process, which mainly includes the following steps: the signal stimulus, then autophagosome formation and fusion with lysosomes, and finally the degradation and release of its contents.

It has also been copiously reported that autophagy can facilitate the renewal of cellular constituents to guarantee energy and materials of quality needed to sustain metabolic reactions, which orchestrates such biological processes as proliferation and differentiation of cells under various physiological or pathological conditions [57]. Typically, autophagy exists at a low level and a basal rate in most cells [58], but it can be activated rapidly in response to excessive release of ROS, abnormal aggregates of misfolded proteins, or a collapse of mitochondrial membrane potential (MMP) apart from infection, cancer, ATP, or nutrient deficiency [59, 60].

It is well established that only adequate autophagy is a kind of cellular self-defense mechanism in times of oxidative stress and other unfavorable conditions [61]. However, improper autophagy above or below a certain threshold is instead disadvantageous [11], likely accelerating the progression of all the related diseases such as neurodegenerative diseases, cerebral ischemia, and cancer [62, 63].

### 3.1. Autophagy-Relative Marker Proteins

As discussed previously, autophagic elimination is a highly sophisticated process during which unwanted or redundant organelles and bits of cytoplasm are enveloped then sweeped away in a lysosome-dependent manner. Each step is finely regulated by relevant proteins that were first discovered in yeast but later verified in higher organisms [63, 64].

LC3 is a mammalian, homologous protein of Atg8 in yeast that has been identified to be the most widely used specific marker of autophagy initiation. LC3 is first synthesized as its precursor, then cut up into its cytosolic form, LC3-I, which can be processed into LC3-II [65]. LC3-II specifically binds to the newly formed autophagosome essential for the elongation stage of the phagophore membrane. The amount of autophagosome can be mirrored by the expression of LC3-II or LC3-II/LC3-I [66]. Mizushima et al. [67] were able to dynamically trace the formation of autophagosomes by using fluorescence characteristics of GFP in established GFPLC3 transgenic mice, which greatly facilitated the study of the molecular mechanisms of autophagy. Beclin1, the first mammalian autophagy-related gene to be identified, regulates the activity of autophagy particularly in the initiation phase by combining with different ligands [68]. Beclin1 can modulate autophagic flux by interacting with PINK1 [69].

In addition, there are observable changes of p62/SQSTM1 in the progression of canonical autophagy [70]. P62 is negatively correlated with autophagy activity, reflecting the degradative capability of autophagy and the intensity of autophagic flux [71]. The receptor protein p62 can be recruited to the autophagosome membrane when LC3-interacting region (LIR) motif targets a substrate (ubiquitinated protein aggregates, damaged mitochondria [72]) and initiates selective degradation in an autophagy-lysosome manner.

### 3.2. Autophagy-Relative Signal Pathway

Prevailing studies indicate that signal transduction pathways associated with autophagy may be more complex than the following two: the mammalian target of rapamycin (mTOR) pathway and the class III phosphatidylinositol 3-kinase (PI3K-III) complex.

Mammalian target of rapamycin (mTOR), a serine/threonine protein kinase, is engaged in autophagy modulation as a dominant downstream negative regulator [73]. mTOR complexes exist in two types, namely mTORC1 and mTORC2, which are distinguished by different components. mTORC1, a regulatory associated protein composed of Rictor, has been demonstrated to terminate the autophagy progression as a critical signaling molecule that is susceptible to the strong inhibition of rapamycin [74, 75]. When cells suffer hypoxia, energy depletion, and other stimuli, mTORC1 activity is simultaneously restrained with the activation of autophagy. Suppressed mTORC1 plays a causal role in the activation of ULK1 complex by dephosphorylating the autophagy-related gene13 (Atg13) and mediating a tighter combination of ULK1, Atg13, and FIP200. ULK1 is homologous with Atg1 in yeast, which has been found to be involved in the induction of autophagy. The ULK1-Atg13-FIP200 complex is not only a direct target of mTOR but a key regulator of other autophagy-related signaling pathways.

The PI3K-III complex is composed of VPS34 (catalytic subunit), Beclin1, and Atg14. When activated by the ULK1 complex, the PI3K-III complex is positioned into the endoplasmic reticulum and further generates PI3P that binds to downstream effectors, playing an important role in the earlier period of autophagic vacuole formation [76, 77]. When discussing the PI3K-III complex, it is common to mention that the class I PI3K and its downstream target AKT, as with MAPK/ERK1/2 signaling, which can exert negative regulatory effects at any stage of induction of autophagy via activating mTOR [78].

**Figure 2:**
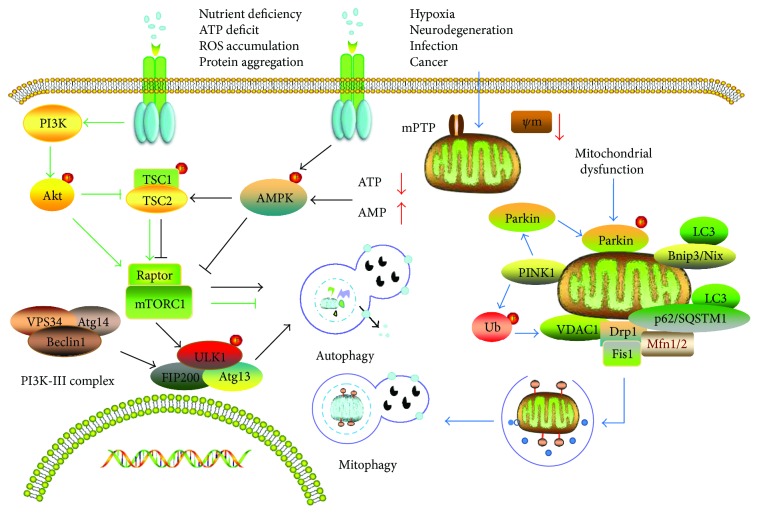
The generation and scavenging of reactive oxygen species (ROS) in mitochondria. The “→” refers to activation or induction, and the “⊢” refers to inhibition. Under normal or stress conditions, ROS is mainly born from the mitochondrial respiratory chain with the beginning of O_2_^−^ production, followed by the conversion to H_2_O_2_ then OH^−^ under the catalysis of SOD and GSH-px. Defective mitochondria can instigate ROS accumulation with a “RIRR” positive-feedback mechanism. Excessive ROS can inflict severe damage on biomacromolecules, which can be counteracted by the antioxidant enzyme system to some degree.

Arguably, distinct signaling pathways involved in the autophagic process vary with different adverse stimuli. AMP-dependent protein kinase (AMPK), an upregulated modulator of autophagy, can sense subtle levels of ATP. On the one hand, AMPK can activate autophagy with a direct inhibitory effect on mTORC1 [79]. On the other hand, p-AMPK can activate TSC1-TSC2 complex, indirectly suppressing the activity of mTORC1 and concurrently initiating autophagy [80]. In addition, AMPK can also combine with ULK1 complex and phosphorylate ULK1, accelerating the progress of autophagic membrane formation [81] (Figure 2).

### 3.3. Mitophagy

Past studies have argued that autophagy does not select which substrates are to be degraded [82]. However, a widely accepted view, proposed in 2005, is that there is a selective form of autophagy in which damaged or unnecessary mitochondria are eliminated [83]. This nonclassical autophagy was defined as mitophagy, and simultaneously or successively, other types of selective autophagy such as xenophagy, pexophagy, ribophagy, and reticulophagy were also identified [59, 84].

Mitochondria are a sensitive organelle ubiquitously found in eukaryotic cells. They are responsible not only for energy-generating processes, but also for producing a basic amount of ROS [85]. Mitochondria form a complicated network regulated by other cellular mechanisms, in which mitochondria are interconnected and interlocked in a perfectly coordinated order. Impaired mitochondria are a threat to proper cellular function because they result in a lack of energy generation and excessive release of ROS [86]. Therefore, it is urgent that dysfunctional mitochondria that interfere with the energy supply and provoke oxidative stress be quickly removed [87]. Fortunately, mitophagy can shoulder this responsibility as an effective cytoplasmic protection mechanism.

Mitophagy is a programmed mitochondrial elimination mechanism that fosters a balance of mitochondrial quantity and quality [59, 87]. It usually occurs in the case of an abnormal increase of ROS, poor nutrition, hypoxia [88], cells senescence, and such stress. These stimuli can cause mitochondrial membrane depolarization or a loss of MMP. Pathological opening of the mPTP may serve as the switch for mitophagy. Existing studies suggest that there are two relatively recognized mitophagy pathways involved in mitochondrial homeostasis. These two pathways are the PINK1/Parkin-mediated pathway and the Bnip3/Nix-mediated pathway. The PINK1/Parkin-mediated pathway is closely associated with Parkinson's disease and is a topic of current research [89].

#### 3.3.1. PINK1/Parkin-Mediated Pathway

PINK1, a serine/threonine protein kinase, is located on the outer membrane of mitochondria and is the upstream regulator of Parkin [90]. Parkin is an E3 ubiquitin ligase, which is present in the cell plasma [91] but has no mitochondrial targeting sequence (MTS) [92]. As a matter of fact, PINK1 can be degraded away quickly by proteolytic enzymes in healthy mitochondria. In the disturbed mitochondria, it will accumulate following depolarization of the membrane potential, phosphorylate Parkin, and then recruit Parkin from the cytoplasm [90]. Along with strengthening E3 ubiquitin ligase activity, Parkin can ubiquitinate the mitochondrial matrix proteins (voltage-dependent anion-selective channel protein 1, VDAC1), recruit p62/SQSTM1 to the surface of mitochondria, and then combine with LC3 to initiate mitophagy [93].

Emerging research indicates that RAD6A (Ube2a), a gene encoding ubiquitin binding enzyme (E2) that is required for the ubiquitination and subsequent clearance of defective mitochondria, can operate with Parkin to regulate mitophagy upon mitochondrial depolarization in mice cortical neurons. Whether the program is dependent on PINK1 needs further scrutiny [94].

#### 3.3.2. Bnip3/Nix-Mediated Mitophagy

Bnip3, a proapoptotic protein, has some degree of homogeneity with BCL-2. Nix is 56% homologous with Bnip3. Both widely existed in mitochondria and are implicated with autophagy and mitophagy in particular [95]. Bnip3 induces autophagy after hypoxic damage and has been reported to have a protective effect by removing injured mitochondria [96]. Recent studies have shown that Bnip3/Nix directly interacts with LC3 to activate the mitophagy pathway [97, 98]. Some researchers believe that though Bnip3 and Nix are involved in mitophagy upon the loss of mitochondrial membrane potential, they may execute mitochondrial clearance via independent but functionally related mechanisms [99, 100] (Figure 2).

Additionally, Mieap can also induce mitophagy after ROS and oxidative damage to restore a healthy pool of mitochondria [101]. Last but not least, mitochondrial fusion, division, and transportation are tightly linked to mitophagy [102].

### 3.4. Autophagy in Neurological Disorders

Not surprisingly, autophagy is extensively observed in nervous system disorders [61]. It has long been thought that autophagy is the primary means for the biodegradation of abnormal protein aggregation and dysfunctional organelles in CI, AD, and PD [103]. Defects in mitochondrial autophagy will aggravate ischemic tissue damage with irreversible neurologic deficit [104], render cognitive and memory defects in AD as a consequence of progressive aggregation of A*β* [105], and promote dopaminergic neuronal death and the occurrence of PD [106]. These results of defects in mitochondrial autophagy indicate that mitophagy acts as an endogenous protective mechanism in the process of neurological disorders. At present, although a growing number of studies have argued that autophagy is activated in various rat and mouse models of cerebral ischemia or hypoxia-ischemia [9, 107–110], whether autophagy is protective or detrimental in the process of CI still remains unclear [111].

## 4. Reactive Oxygen Species (ROS) and Autophagy

A growing body of reports has demonstrated that most stressful events, such as nutrient deficit and hypoxia, which necessitate a greater energy supply and then aggravate mitochondrial burden along with increasing ROS, are related to the initiation of autophagy [16]. Intriguingly, an increasing amount of evidence suggests that ROS are seen as essential signals to activate autophagy under various stimulating conditions [112, 113]. Both moderate and increased ROS levels can specifically trigger mitophagy which is conducive to cell survival in a different manner, while only excessive ROS can activate general autophagy [114].

The molecular signaling pathways involved in both the initiation and execution of autophagy following exposure to ROS are sophisticated [16, 18]. The pathways mainly include transcriptional progress in the nucleus and posttranscriptional progress in the cytoplasm. These specific transcriptional regulatory mechanisms first involve the activation of HIF-1, p53, FOXO3, and NRF2; then, the corresponding proteins are produced and modulation of autophagy occurs where the cytoplasm was exposed to ROS. Take hypoxia-inducible factor (HIF) for example, it is involved in cell survival under hypoxic conditions and participates in the transcription of Bnip3 and NIX in response to ROS. These autophagy-associated protein products can constitutively stimulate autophagic clearance of damaged mitochondria and decrease ROS levels [115].

In addition, numerous studies have supported that ROS may regulate autophagy via mTOR-dependent pathways in the cytoplasm [116–118]. Nevertheless, most of the literature maintains that ROS available to elicit autophagy are mainly H_2_O_2_ and O_2_^−^ produced by mitochondria [14, 112]. When there is an elevated level of H_2_O_2_, a relatively stable and prolonged stimuli, suppressed autophagy via the PI3K-Akt pathway, can be reactivated by blocking PTEN as well as inhibiting the activity of Akt or mTORC1 [119]. Similarly, H_2_O_2_ in excess can induce autophagy in an AMPK-dependent manner and is accompanied by the decline of mTORC1 activity [18]. Beyond that, a wide range of stress response proteins such as p38MAPK, extracellular regulated kinase (ERK), and c-Jun N-terminal kinase (JNK) is also involved with autophagy induction in the presence of abundant ROS [120]. Taken together, it is an indisputable fact that ROS is an available regulator despite autophagy making a difference in both cell survival and death as a double-edged sword.

From another perspective, autophagy has been proposed as a potential survival mechanism in the face of ROS production by removing damaged or redundant components to prevent unnecessary oxidative damage [19]. Furthermore, there are increased intracellular ROS levels in cells with defective autophagy protein Atg7 [121]. Specially, the selective elimination of dysfunctional mitochondria via autophagy also serves as a cytoprotective process to limit the production of ROS and avoid potential oxidant injury [122].

It is also believed that a number of signal transduction pathways related to autophagy are available to modulate ROS. The Keap1-Nrf2 system is now considered a defense mechanism upon exposure to oxidative stress [123, 124]. As mentioned earlier, the p62/SQSTM1 protein, or p62 for short, may contribute to autophagosome formation as an autophagic adaptor and/or receptor [125]. Phosphorylation of P62 in the mTORC1-dependent autophagy pathway can promote the integration of ubiquitinated cargos and phosphorylated Keap1, which is necessary for the degradation of Nrf2 [126, 127]. Released Nrf2 is reactivated, translocated into the nucleus while binding to ARE, and eventually stimulates transcription of antioxidant genes. Beyond that, mitochondrial hexokinase II (HKII) shares a deep relationship with autophagy and redox homeostasis. HKII induces the inactivation of mTORC1, further opens mPTP, and creates a preventive antioxidant defense by decreasing release of ROS [128, 129].

**Figure 3:**
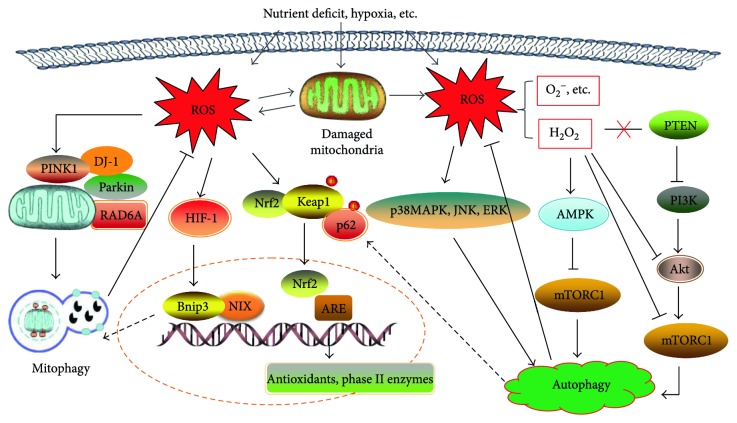
The interrelation of ROS and autophagy/mitophagy, coupled with the relevant signal transduction pathways. ROS available to induce autophagy is mainly mitochondrial H_2_O_2_ and O_2_^−^, which may modulate autophagy via mTOR-dependent pathways. ROS-induced autophagy and mitophagy both can abort ROS for redox homeostasis. In response to abundant ROS, the Keap1/Nrf2/ARE cascade is activated as a potent antioxidant mechanism. Phosphorylation of P62 by autophagy can promote the integration of phosphorylated Keap1 and ubiquitinated Nrf2, then negative regulation of Keap1 frees Nrf2 from degradation, and reactivated Nrf2 is translocated into the nucleus to bind to ARE for the transcription of antioxidant genes and phase II enzymes.

In conclusion, there is little doubt that ROS play a positive role in the activation of autophagy under various stimulating conditions [112, 113]. By coincidence, autophagy plays a crucial role in maintaining redox homeostasis [6]. ROS can induce autophagy, and autophagy serves as a buffer system to control the level of ROS in cells and reduce their toxic effects [130]. The interplay of autophagy and redox response via various signaling pathways may be involved with the modulation of cellular homeostasis [127] (Figure 3).

### 4.1. Reactive Oxygen Species (ROS) and Mitophagy

As stated earlier, mitochondria are believed to be the primary source of ROS. Coincidentally but unfortunately, they are also the major target of oxidative stress triggered by ROS, which may result from the fact that mitochondria are an important site for nucleic acid, lipid, and amino acid production. Excessive ROS stimuli can inflict peroxidation damage on these biomacromolecule precursors and create toxic byproducts [131]. Note that mtDNA lacks the protection of histones, and its repair capacity is rather poor. It is therefore more vulnerable to ROS than nuclear DNA [132] and is bound to leave mitochondria heavily damaged by ROS.

Mitochondrial dysfunction caused by a high concentration of ROS not only can activate and regulate nonselective autophagy, but also can be involved in mitophagy which selectively removes damaged mitochondria. ROS and oxidative stress have been shown to be involved in the recruitment and localization of Parkin and DJ-1, specific proteins that are closely tied to the activation of mitophagy [133].

Selective autophagy is a protective mechanism that reduces ROS production by means of removing unneeded mitochondria, thereby alleviating oxidative damage [16, 122]. More importantly, defects in mitophagy can aggravate lipotoxicity, hinder selective degradation of defective mitochondria caused by ROS, and thus cause subsequent damage to the cells [134].

Haddad et al. [94] discovered that RAD6A can cooperate with Parkin to ubiquitinate mitochondrial proteins associated with the initiation of mitophagy for clearing dysfunctional mitochondria and dampening oxidative stress. Particularly, RAD6A mutations cause neuronal function defects primarily by disrupting mitophagy (Figure 3).

### 4.2. Reactive Oxygen Species (ROS) and Autophagy (Mitophagy) in Neurological Disorders

ROS are described as the culprit of almost all neurological conditions [135]. Mounting evidence has indicated that ROS participate actively in autophagy in many cells, including neurons [131, 136]. Autophagy removes or degrades nonfunctional cytoplasmic content as an intracellular self-purification mechanism. Neurons are highly sensitive to autophagic degradation, and the integrity of mature neurons depends on the high level of autophagy because of their postmitotic nature [14, 137]. Also, autophagy can reduce ROS damage by eliminating unnecessary or damaged organelles and abnormal protein aggregates, as well as inhibiting the excessive activation of ROS in response to neuronal damage, which is conducive to the survival of nerve cells [138]. Emerging evidence indicates that autophagy may exhibit an antioxidant defense system, which has been proposed to provide a remarkable impact on neuronal bioenergetic health [139].

#### 4.2.1. Reactive Oxygen Species (ROS) and Autophagy (Mitophagy) in CI

Ischemic cerebrovascular disease (CI) is a leading cause of death and disability worldwide [140]. Currently, endovascular intervention and venous thrombolysis are conventional therapies for restoring the blood supply required for the recovery of nervous function. However, both animal studies and clinical findings have revealed that reperfusion following ischemia results in a more serious brain damage [141]. The ischemia-reperfusion injury is a complicated pathological process involving multiple factors, among which oxidative stress stands out [5, 142]. There is an extensive damage of mitochondria, including irregular mitochondria swelling and their crista fragmenting, in CI especially during the acute phase. This damage stimulates mPTP to open continuously, leading to a change in membrane potential, energy deficit, and ROS generation, thus inducing autophagy.

It has been observed that autophagy occurs dramatically in the mouse striatum and cortex following cerebral hypoxic-ischemic injury and can then be strongly amplified by an ensuing overproduction of ROS. Autophagy in this context can substantially rescue neurons in the ischemic penumbra by preventing necrosis and apoptosis via eliminating impaired mitochondria [143].

It has been reported that FA deficiency dramatically alters ischemia-induced activation of autophagy. This is reflected by the elevated levels of LC3 and Beclin1 expression, which are accompanied by a remarkable increase in 8-OHdG, indicating that FA deficiency may enhance autophagy levels by triggering oxidative damage [144]. One study has shown that both ROS and autophagy are engaged in reperfusion injury after cerebral ischemia and that autophagy can be activated by antioxidants. The application of antioxidants or autophagy revulsive can reduce neuronal damage and significantly decrease the infarction area [145]. Thus, we can speculate that antioxidants might play a protective role in ischemic injury by inducing autophagy. There may be some more complicated mechanisms of crosstalk between autophagy and oxidative stress in need of further research. One study pointed to the finding that ischemic insults could immediately activate autophagy as a neuroprotective mechanism, which significantly affects ROS generation and oxidative toxicity. As well, pharmacological inhibition of autophagy or lysosomes can delay the mitochondrial ROS burst [146]. Scherz-Shouval and Elazar [15] have argued that ROS can upregulate autophagy through multiple signaling pathways. Sirt3 is a conserved deacetylase associated with biological functions such as energy metabolism, stress resistance, and mitochondrial redox homeostasis. Furthermore, it can positively regulate autophagy through the AMPK-mTOR pathway [147], which promotes neuronal survival within an in vitro oxygen and glucose (OGD) deprivation model of cerebral ischemia created by attenuating H_2_O_2_ and O_2_^−^ [148]. Pharmacological or genetic inhibition of autophagy can ameliorate SIRT6-mediated neuronal injury, probably via attenuating AKT signaling closely related to oxidative stress in the OGD model of SH-SY5Y neurons [149]. Further, in vivo mechanistic studies are needed to verify the interplay of oxidative stress and autophagy. Furthermore, moderate activation of ROS can promote the translocation of Parkin to injured mitochondria and then incur Parkin-mediated mitophagy and ensure the integrity of mitochondria in ischemic brain injury [150] (Table 1).

#### 4.2.2. Reactive Oxygen Species (ROS) and Autophagy (Mitophagy) in AD

Alzheimer's disease (AD) is one of the most common types of late-onset neurodegenerative diseases, hallmarked by a progressive loss of memory and cognition coupled with typical pathological features including neuritic plaques (NPs) and neurofibrillary tangles (NFTs) [46, 151]. Enhanced ROS and oxidative damage have been proven to be implicated in the evolution of neuronal dysfunction during the early events of AD [152].

Growing evidence suggests that spatial learning and memory deficits in AD may be tightly correlated with the impairment of the Nrf2-ARE pathway since Nrf2 knockout confers AD model mice with more sensitivity to neuronal damage. Strikingly, some scholars have proposed that the interaction between oxidative stress and mitochondrial dysfunction may be involved in the process of AD because of the influence oxidative stress has on mitochondrial transport [153]. It has been observed that autophagic vacuoles with engulfed, defective mitochondria increased in the pyramidal neurons of AD patients [7]. The autophagic removal of damaged mitochondria and A*β* mediated by Parkin can attenuate oxidative stress and restore the energy supply so as to delay or prevent neurodegeneration in AD transgenic mice [105] (Table 1).

#### 4.2.3. Reactive Oxygen Species (ROS) and Autophagy (Mitophagy) in PD

Parkinson's disease (PD) is a movement disorder with three outstanding clinical characteristics: bradykinesia, resting tremor, rigidity, and postural instability. Although the underlying etiology of PD is still far from clear, oxidative stress and mitophagy deficiency have been proposed as the principal elements in the development of dopaminergic neuronal death in the substantia nigra of PD patients.

Neurodegenerative diseases such as PD are often accompanied by increased oxidative brain damage coinciding with a reduction in antioxidants. This leads to dysfunctional mitochondria or protein aggregates that can be rescued to some extent by radical scavengers. Autophagy has been proposed as an endogenous, antioxidant, protective pathway that can clear accumulated ROS and reverse established ROS-induced protein, DNA, and lipid damage independent of the disposal of radical scavengers [154]. Protein accumulation and oxidative stress are pathologically pronounced in neurodegenerative diseases. Enhancing autophagy could scavenge aggregate-prone proteins and increased ROS, while antioxidants could block the benefits of autophagy and exacerbate neurodegeneration [155].

Mitochondria have a central role in redox regulation of autophagy as the generator and scavenger of ROS [156], but can be attacked when ROS exceed the scavenging activity. The dysfunction of mitochondria is a prominent initiating factor of nervous system diseases [157]. This dysfunction then amplifies oxidative damage, with the underlying assumption that the quality and quantity of mitochondria significantly affects neuronal function. Mitophagy was originally proposed to clear disturbed mitochondria after pathological stress in an attempt to restore homeostasis [158].

Dagda et al. discovered that knockdown of PINK1 in a recessive PD model can result in the accumulation of mitochondrial ROS, accompanied by clustered fragmented mitochondria and depolarized mitochondria which correlate with autophagy. More importantly, autophagy does play an essential role in limiting dopaminergic neuronal death in this genetic model and RNAi knockdown of genes necessary for inducing autophagy exacerbates the occurrence of PD [159] (Table 1).

Several studies have claimed that mitochondrial dysfunction and the existence of mitochondrial complex *Ι* defects also contribute immeasurably to the disease by playing a causative or consequential role in the exacerbation of oxidative stress in dopaminergic neurons. DJ-1, a causative protein of familial PD, is essential for modulating PINK/Parkin-mediated mitophagy [160]. Both DJ-1 and DJ-1-binding compounds have been identified as neuroprotective against oxidative stress in PD rats [161].

## 5. Conclusion and Perspective

Plenty of studies have repeatedly shown that ROS accumulation displays detrimental implications for the basic function and survival of neurons. ROS or oxidative stress can provoke autophagy, and autophagy can take part in the removal and repair of ROS-induced oxidative lesions through a variety of signaling pathways. But autophagic neuronal death will still result if cumulative ROS go beyond the scavenging activity of autophagy. At present, it appears to be contradictory that autophagy serves as a cellular self-purification mechanism, but hyperactivity or hypoactivity of autophagy is unfavorable for the normal functionality of neurons [162, 163]. After all, the predetermined threshold level of perfect autophagy is often blurred, particularly under a variety of disease courses. So, more relevant, constructive research should be undertaken without delay.

Mitochondria are thought to be crucial for neuronal function and fate by supplying energy and modulating redox status. It is well established that brain mitochondrial dysfunction or mitophagy defects are strongly associated with the initiation and progression of CI, AD, and PD. Neuronal mitochondrial impairments exhibit pronounced effects on mitochondrial membrane potential, leading to the prolonged opening of mPTP and an elevated production of ROS which can be rescued by mitophagy and ensuing mitochondrial turnover. Concurrently, treatment with mitochondria-targeted antioxidants substantially mitigates neuronal mitochondrial disturbance and oxidative damage [164, 165].

In summary, we provided a basic knowledge of ROS and autophagy/mitophagy and then expatiated specifically on the interrelation between ROS and autophagy as well as on their molecular regulatory mechanisms. Finally, we discussed the interplay of ROS and autophagy in CI, AD, and PD. Nonetheless, a lot of current work only focuses on the close interplay between ROS and autophagy/mitophagy in CI and PD, while there are few studies on how they are involved in AD and the underlying, precise, regulatory mechanisms are not well investigated. In the future, more basic research is needed to further excavate the correlation between autophagy/mitophagy and ROS together with their possible mechanisms in neurological disorders. Such research will lay a good foundation for pinpointing late-model drug targets and exploring aggressive therapeutic tactics that are applicable for the clinical treatment of such life-threatening neurological diseases.

## Figures and Tables

**Table 1:** The interplay between ROS and autophagy/mitophagy in neurological diseases.

Author	Year	Model (animal/cell)	Main idea	Effect of autophagy
Zhao et al. [144]	2016	MCAO/SD rats	FA deficiency simultaneously enhanced the activity of autophagy and induced the generation of oxidative stress following the MCAO model; oxidative injury seems to be involved in excessive activation of autophagy caused by FA deficiency.	Detrimental

Wenjing et al. [145]	2013	Mouse & neural cells	Autophagy is upregulated, and the level of ROS is elevated in the central nervous system after ischemia-reperfusion; Antioxidants can protect neural cells and decrease infarct volume possibly by activating the autophagic pathway of cells.	Protective

Kubota et al. [146]	2010	MCAO/SD rats	Chemical inhibitors of autophagy or lysosomes can delay the release of mitochondrial ROS to prolong the therapeutic time window. Ischemic insults will immediately initiate autophagy induction with undefined mechanisms, which significantly will impact ROS production and oxidative damage in vivo.	Detrimental

Dai et al. [147]	2017	OGD/cortical neurons	Sirt3 showed a protective role in eliminating intracellular H_2_O_2_, attenuating mitochondrial O_2_^−^, and promoting autophagy through the AMPK-mTOR pathway in neuronal ischemia.	Protective

Shao et al. [149]	2016	SH-SY5Y/neuronal cells	SIRT6-mediated autophagy contributes to oxidative stress-induced neuronal injury since inhibition of autophagy could prevent the detrimental effect of SIRT6 on cell survival, which could be attributed to attenuation of AKT signaling closely related to oxidative stress.	Detrimental

Khandelwal et al. [105]	2011	3xTg-AD mice	The autophagic removal of A*β* mediated by Parkin can attenuate oxidative stress and mitochondrial dysfunction to restore energy supply for a better modulation of autophagy in AD transgenic mice.	Protective

Giordano et al. [154]	2014	PD mouse model	Autophagy is proposed as an antioxidant protective pathway that can clear cumulative ROS and reverse established ROS-induced protein, DNA, and lipid damage independent of the disposal of radical scavengers.	Protective

Underwood et al. [155]	2010	Mouse cortical neurons	Autophagy can scavenge aggregate-prone proteins and increased ROS, while antioxidants can block autophagy and thereby counterbalance the benefits of autophagy and exacerbate neurodegeneration.	Protective

Dagda et al. [159]	2009	PD cell model/SH-SY5Y	Loss of PINK1 function can stir oxidative stress, which can then elicit coordinated autophagy and mitophagy for mitochondrial turnover by a removal of dysfunctional mitochondria.	Protective

## References

[1] Siti H. N., Kamisah Y., Kamsiah J. (2015). The role of oxidative stress, antioxidants and vascular inflammation in cardiovascular disease (a review). *Vascular Pharmacology*.

[2] Huang J., Lam G. Y., Brumell J. H. (2011). Autophagy signaling through reactive oxygen species. *Antioxidants & Redox Signaling*.

[3] Dröge W. (2002). Free radicals in the physiological control of cell function. *Physiological Reviews*.

[4] Skowronska M., Albrecht J. (2013). Oxidative and nitrosative stress in ammonia neurotoxicity. *Neurochemistry International*.

[5] Chan P. H. (2001). Reactive oxygen radicals in signaling and damage in the ischemic brain. *Journal of Cerebral Blood Flow & Metabolism*.

[6] Levonen A. L., Hill B. G., Kansanen E., Zhang J., Darley-Usmar V. M. (2014). Redox regulation of antioxidants, autophagy, and the response to stress: implications for electrophile therapeutics. *Free Radical Biology & Medicine*.

[7] Moreira P. I., Siedlak S. L., Wang X. (2007). Increased autophagic degradation of mitochondria in Alzheimer disease. *Autophagy*.

[8] Palikaras K., Tavernarakis N. (2012). Mitophagy in neurodegeneration and aging. *Frontiers in Genetics*.

[9] Yan W., Zhang H., Bai X., Lu Y., Dong H., Xiong L. (2011). Autophagy activation is involved in neuroprotection induced by hyperbaric oxygen preconditioning against focal cerebral ischemia in rats. *Brain Research*.

[10] Baehrecke E. H. (2003). Autophagic programmed cell death in *Drosophila*. *Cell Death & Differentiation*.

[11] Clarke P. G. H., Puyal J. (2012). Autophagic cell death exists. *Autophagy*.

[12] Cross C. E., Halliwell B., Borish E. T. (1987). Oxygen radicals and human disease. *Annals of Internal Medicine*.

[13] Diebold L., Chandel N. S. (2016). Mitochondrial ROS regulation of proliferating cells. *Free Radical Biology & Medicine*.

[14] Scherz-Shouval R., Shvets E., Fass E., Shorer H., Gil L., Elazar Z. (2007). Reactive oxygen species are essential for autophagy and specifically regulate the activity of Atg4. *The EMBO Journal*.

[15] Scherz-Shouval R., Elazar Z. (2011). Regulation of autophagy by ROS: physiology and pathology. *Trends in Biochemical Sciences*.

[16] Filomeni G., De Zio D., Cecconi F. (2015). Oxidative stress and autophagy: the clash between damage and metabolic needs. *Cell Death & Differentiation*.

[17] Mi Y., Xiao C., Du Q., Wu W., Qi G., Liu X. (2016). Momordin Ic couples apoptosis with autophagy in human hepatoblastoma cancer cells by reactive oxygen species (ROS)-mediated PI3K/Akt and MAPK signaling pathways. *Free Radical Biology & Medicine*.

[18] Scherz-Shouval R., Shvets E., Elazar Z. (2007). Oxidation as a post-translational modification that regulates autophagy. *Autophagy*.

[19] Kiffin R., Bandyopadhyay U., Cuervo A. M. (2006). Oxidative stress and autophagy. *Antioxidants & Redox Signaling*.

[20] Murphy M. P. (2009). How mitochondria produce reactive oxygen species. *Biochemical Journal*.

[21] Zorov D. B., Juhaszova M., Sollott S. J. (2014). Mitochondrial reactive oxygen species (ROS) and ROS-induced ROS release. *Physiological Reviews*.

[22] Zorov D. B., Filburn C. R., Klotz L. O., Zweier J. L., Sollott S. J. (2000). Reactive oxygen species (ROS-induced) ROS release. *The Journal of Experimental Medicine*.

[23] Bernardi P., Rasola A., Forte M., Lippe G. (2015). The mitochondrial permeability transition pore: channel formation by F-ATP synthase, integration in signal transduction, and role in pathophysiology. *Physiological Reviews*.

[24] Kwak H. J., Liu P., Bajrami B. (2015). Myeloid cell-derived reactive oxygen species externally regulate the proliferation of myeloid progenitors in emergency granulopoiesis. *Immunity*.

[25] Hekimi S., Lapointe J. (2011). Taking a “good” look at free radicals in the aging process. *Trends in Cell Biology*.

[26] Amer J., Goldfarb A., Fibach E. (2003). Flow cytometric measurement of reactive oxygen species production by normal and thalassaemic red blood cells. *European Journal of Haematology*.

[27] Warner D. S., Sheng H., Batinić-Haberle I. (2004). Oxidants, antioxidants and the ischemic brain. *Journal of Experimental Biology*.

[28] Marí M., Morales A., Colell A., García-Ruiz C., Kaplowitz N., Fernández-Checa J. C. (2013). Mitochondrial glutathione: features, regulation and role in disease. *Biochimica et Biophysica Acta (BBA) - General Subjects*.

[29] Schmidley J. W. (1990). Free radicals in central nervous system ischemia. *Stroke*.

[30] Hagar H., Al Malki W. (2014). Betaine supplementation protects against renal injury induced by cadmium intoxication in rats: role of oxidative stress and caspase-3. *Environmental Toxicology and Pharmacology*.

[31] Fink R. C., Scandalios J. G. (2002). Molecular evolution and structure–function relationships of the superoxide dismutase gene families in angiosperms and their relationship to other eukaryotic and prokaryotic superoxide dismutases. *Archives of Biochemistry and Biophysics*.

[32] Rudnicki M., Silveira M. M., Pereira T. V. (2007). Protective effects of *Passiflora alata* extract pretreatment on carbon tetrachloride induced oxidative damage in rats. *Food and Chemical Toxicology*.

[33] Cojocaru I. M., Botezat M., Lazar L., Oprisan A. (2005). Evaluation of oxidative stress in patients with acute ischemic stroke: P2038. *European Journal of Neurology Supplement*.

[34] Rodrigo R., Libuy M., Feliú F., Hasson D. (2013). Oxidative stress-related biomarkers in essential hypertension and ischemia-reperfusion myocardial damage. *Disease Markers*.

[35] Kwok J. B. J., Hallupp M., Loy C. T. (2005). *GSK3B* polymorphisms alter transcription and splicing in Parkinson’s disease. *Annals of Neurology*.

[36] Zenkov N. K., Menshchikova E. B., Tkachev V. O. (2013). Keap1/Nrf2/ARE redox-sensitive signaling system as a pharmacological target. *Biochemistry*.

[37] Sabens Liedhegner E. A., Gao X. H., Mieyal J. J. (2012). Mechanisms of altered redox regulation in neurodegenerative diseases—focus on S-glutathionylation. *Antioxidants & Redox Signaling*.

[38] Guangpin C., Ping Q. (2016). ROS mediated inflammation and neurological diseases in central nervous system. *Chinese Journal of Histochemistry and Cytochemistry*.

[39] Terman A., Brunk U. T. (2005). Autophagy in cardiac myocyte homeostasis, aging, and pathology. *Cardiovascular Research*.

[40] Mariani E., Polidori M. C., Cherubini A., Mecocci P. (2005). Oxidative stress in brain aging, neurodegenerative and vascular diseases: an overview. *Journal of Chromatography B*.

[41] Milhavet O., Lehmann S. (2002). Oxidative stress and the prion protein in transmissible spongiform encephalopathies. *Brain Research Reviews*.

[42] Abramov A. Y., Scorziello A., Duchen M. R. (2007). Three distinct mechanisms generate oxygen free radicals in neurons and contribute to cell death during anoxia and reoxygenation. *Journal of Neuroscience*.

[43] Sharma T., Airao V. (2014). Solasodine protects rat brain against ischemia/reperfusion injury through its antioxidant activity. *European Journal of Pharmacology*.

[44] Shah Z. A., Li R. C. (2007). Role of reactive oxygen species in modulation of Nrf2 following ischemic reperfusion injury. *Neuroscience*.

[45] Shih A. Y., Li P., Murphy T. H. (2005). A small-molecule-inducible Nrf2-mediated antioxidant response provides effective prophylaxis against cerebral ischemia *in vivo*. *Journal of Neuroscience*.

[46] Shi Q., Gibson G. E. (2007). Oxidative stress and transcriptional regulation in Alzheimer’s disease. *Alzheimer Disease & Associated Disorders*.

[47] Nicolas G., Bennoun M., Devaux I. (2001). Lack of hepcidin gene expression and severe tissue iron overload in upstream stimulatory factor 2 (*USF2*) knockout mice. *Proceedings of the National Academy of Sciences of the United States of America*.

[48] Smith M. A., Nunomura A., Lee H.-g. (2005). Chronological primacy of oxidative stress in Alzheimer disease. *Neurobiology of Aging*.

[49] Puertas M. C., Martínez-Martos J. M., Cobo M. P., Carrera M. P., Mayas M. D., Ramírez-Expósito M. J. (2012). Plasma oxidative stress parameters in men and women with early stage Alzheimer type dementia. *Experimental Gerontology*.

[50] Sanders L. H., Timothy Greenamyre J. (2013). Oxidative damage to macromolecules in human Parkinson disease and the rotenone model. *Free Radical Biology & Medicine*.

[51] Ramsey C. P., Glass C. A. (2007). Expression of Nrf2 in neurodegenerative diseases. *Journal of Neuropathology & Experimental Neurology*.

[52] Hwang Y. P., Jeong H. G. (2010). Ginsenoside Rb1 protects against 6-hydroxydopamine-induced oxidative stress by increasing heme oxygenase-1 expression through an estrogen receptor-related PI3K/Akt/Nrf2-dependent pathway in human dopaminergic cells. *Toxicology and Applied Pharmacology*.

[53] Ashford T. P., Porter K. R. (1962). Cytoplasmic components in hepatic cell lysosomes. *The Journal of Cell Biology*.

[54] Deter R. L., Baudhuin P., De Duve C. (1967). Participation of lysosomes in cellular autophagy induced in rat liver by glucagon. *The Journal of Cell Biology*.

[55] He C., Klionsky D. J. (2009). Regulation mechanisms and signaling pathways of autophagy. *Annual Review of Genetics*.

[56] Martinez-Vicente M. (2015). Autophagy in neurodegenerative diseases: from pathogenic dysfunction to therapeutic modulation. *Seminars in Cell & Developmental Biology*.

[57] Kroemer G., Mariño G., Levine B. (2010). Autophagy and the integrated stress response. *Molecular Cell*.

[58] Levine B., Kroemer G. (2008). Autophagy in the pathogenesis of disease. *Cell*.

[59] Kim I., Rodriguez-Enriquez S., Lemasters J. J. (2007). Selective degradation of mitochondria by mitophagy. *Archives of Biochemistry and Biophysics*.

[60] Levine B., Klionsky D. J. (2004). Development by self-digestion: molecular mechanisms and biological functions of autophagy. *Developmental Cell*.

[61] Nikoletopoulou V., Papandreou M. E. (2015). Autophagy in the physiology and pathology of the central nervous system. *Cell Death & Differentiation*.

[62] Jiang P., Mizushima N. (2014). Autophagy and human diseases. *Cell Research*.

[63] Parzych K. R., Klionsky D. J. (2014). An overview of autophagy: morphology, mechanism, and regulation. *Antioxidants & Redox Signaling*.

[64] Itakura E., Mizushima N. (2010). Characterization of autophagosome formation site by a hierarchical analysis of mammalian Atg proteins. *Autophagy*.

[65] Tanida I., Ueno T., Kominami E. (2004). LC3 conjugation system in mammalian autophagy. *The International Journal of Biochemistry & Cell Biology*.

[66] Kabeya Y., Mizushima N., Ueno T. (2000). LC3, a mammalian homologue of yeast Apg8p, is localized in autophagosome membranes after processing. *The EMBO Journal*.

[67] Mizushima N., Yamamoto A., Matsui M., Yoshimori T., Ohsumi Y. (2004). In vivo analysis of autophagy in response to nutrient starvation using transgenic mice expressing a fluorescent autophagosome marker. *Molecular Biology of the Cell*.

[68] Wirawan E., Lippens S., Vanden Berghe T. (2012). Beclin1: a role in membrane dynamics and beyond. *Autophagy*.

[69] Michiorri S., Gelmetti V., Giarda E. (2010). The Parkinson-associated protein PINK1 interacts with Beclin1 and promotes autophagy. *Cell Death and Differentiation*.

[70] Li L., Chen J., Sun S., Zhao J., Dong X., Wang J. (2017). Effects of estradiol on autophagy and Nrf-2/ARE signals after cerebral ischemia. *Cellular Physiology and Biochemistry*.

[71] Ichimura Y., Komatsu M. (2010). Selective degradation of p62 by autophagy. *Seminars in Immunopathology*.

[72] Narendra D., Kane L. A., Hauser D. N., Fearnley I. M., Youle R. J. (2010). p62/SQSTM1 is required for Parkin-induced mitochondrial clustering but not mitophagy; VDAC1 is dispensable for both. *Autophagy*.

[73] Jung C. H., Ro S. H., Cao J., Otto N. M., Kim D. H. (2010). mTOR regulation of autophagy. *FEBS Letters*.

[74] Benjamin D., Colombi M., Moroni C., Hall M. N. (2011). Rapamycin passes the torch: a new generation of mTOR inhibitors. *Nature Reviews Drug Discovery*.

[75] Yuan H. X., Russell R. C., Guan K. L. (2013). Regulation of PIK3C3/VPS34 complexes by MTOR in nutrient stress-induced autophagy. *Autophagy*.

[76] Nazio F., Cecconi F. (2013). mTOR, AMBRA1, and autophagy: an intricate relationship. *Cell Cycle*.

[77] Yang Z., Klionsky D. J. (2010). Mammalian autophagy: core molecular machinery and signaling regulation. *Current Opinion in Cell Biology*.

[78] Petiot A., Ogier-Denis E., Blommaart E. F. C., Meijer A. J., Codogno P. (2000). Distinct classes of phosphatidylinositol 3′-kinases are involved in signaling pathways that control macroautophagy in HT-29 cells. *Journal of Biological Chemistry*.

[79] Inoki K., Ouyang H., Zhu T. (2006). TSC2 integrates Wnt and energy signals via a coordinated phosphorylation by AMPK and GSK3 to regulate cell growth. *Cell*.

[80] Alers S., Loffler A. S., Wesselborg S., Stork B. (2012). Role of AMPK-mTOR-Ulk1/2 in the regulation of autophagy: cross talk, shortcuts, and feedbacks. *Molecular and Cellular Biology*.

[81] Kim J., Kundu M., Viollet B., Guan K. L. (2011). AMPK and mTOR regulate autophagy through direct phosphorylation of Ulk1. *Nature Cell Biology*.

[82] Kopitz J., Kisen G. O., Gordon P. B., Bohley P., Seglen P. O. (1990). Nonselective autophagy of cytosolic enzymes by isolated rat hepatocytes. *The Journal of Cell Biology*.

[83] Lemasters J. J. (2005). Selective mitochondrial autophagy, or mitophagy, as a targeted defense against oxidative stress, mitochondrial dysfunction, and aging. *Rejuvenation Research*.

[84] Beau I., Esclatine A., Codogno P. (2008). Lost to translation: when autophagy targets mature ribosomes. *Trends in Cell Biology*.

[85] Frederick R. L., Shaw J. M. (2007). Moving mitochondria: establishing distribution of an essential organelle. *Traffic*.

[86] Novak I. (2012). Mitophagy: a complex mechanism of mitochondrial removal. *Antioxidants & Redox Signaling*.

[87] Ashrafi G., Schwarz T. L. (2013). The pathways of mitophagy for quality control and clearance of mitochondria. *Cell Death & Differentiation*.

[88] Zhang H., Bosch-Marce M., Shimoda L. A. (2008). Mitochondrial autophagy is an HIF-1-dependent adaptive metabolic response to hypoxia. *Journal of Biological Chemistry*.

[89] Redmann M., Dodson M., Boyer-Guittaut M., Darley-Usmar V., Zhang J. (2014). Mitophagy mechanisms and role in human diseases. *The International Journal of Biochemistry & Cell Biology*.

[90] Thomas R. E., Andrews L. A., Burman J. L., Lin W. Y., Pallanck L. J. (2014). PINK1-Parkin pathway activity is regulated by degradation of PINK1 in the mitochondrial matrix. *PLoS Genetics*.

[91] Matsuda N., Tanaka K., Komatsu M. (2012). Role of mitophagy in hereditary Parkinson’s disease. *Brain and Nerve*.

[92] Springer W., Kahle P. J. (2011). Regulation of PINK1-Parkin-mediated mitophagy. *Autophagy*.

[93] Geisler S., Holmström K. M., Skujat D. (2010). PINK1/Parkin-mediated mitophagy is dependent on VDAC1 and p62/SQSTM1. *Nature Cell Biology*.

[94] Haddad D. M., Vilain S., Vos M. (2013). Mutations in the intellectual disability gene Ube2a cause neuronal dysfunction and impair parkin-dependent mitophagy. *Molecular Cell*.

[95] Zhang J., Ney P. A. (2009). Role of BNIP3 and NIX in cell death, autophagy, and mitophagy. *Cell Death & Differentiation*.

[96] Hamacher-Brady A., Brady N. R., Logue S. E. (2006). Response to myocardial ischemia/reperfusion injury involves Bnip3 and autophagy. *Cell Death & Differentiation*.

[97] Hanna R. A., Quinsay M. N., Orogo A. M., Giang K., Rikka S., Gustafsson Å. B. (2012). Microtubule-associated protein 1 light chain 3 (LC3) interacts with Bnip3 protein to selectively remove endoplasmic reticulum and mitochondria via autophagy. *Journal of Biological Chemistry*.

[98] Novak I., Kirkin V., McEwan D. G. (2010). Nix is a selective autophagy receptor for mitochondrial clearance. *EMBO Reports*.

[99] Elmore S. P., Qian T., Grissom S. F., Lemasters J. J. (2001). The mitochondrial permeability transition initiates autophagy in rat hepatocytes. *The FASEB Journal*.

[100] Twig G., Elorza A., Molina A. J. A. (2008). Fission and selective fusion govern mitochondrial segregation and elimination by autophagy. *The EMBO Journal*.

[101] Kitamura N., Nakamura Y., Miyamoto Y. (2011). Mieap, a p53-inducible protein, controls mitochondrial quality by repairing or eliminating unhealthy mitochondria. *PLoS One*.

[102] Chen H., Chan D. C. (2009). Mitochondrial dynamics–fusion, fission, movement, and mitophagy–in neurodegenerative diseases. *Human Molecular Genetics*.

[103] Mizushima N., Levine B., Cuervo A. M., Klionsky D. J. (2008). Autophagy fights disease through cellular self-digestion. *Nature*.

[104] Zhang X., Yan H., Yuan Y. (2013). Cerebral ischemia-reperfusion-induced autophagy protects against neuronal injury by mitochondrial clearance. *Autophagy*.

[105] Khandelwal P. J., Herman A. M., Hoe H. S., Rebeck G. W., Moussa C. E. H. (2011). Parkin mediates beclin-dependent autophagic clearance of defective mitochondria and ubiquitinated Aβ in AD models. *Human Molecular Genetics*.

[106] Feng D., Liu L., Zhu Y., Chen Q. (2013). Molecular signaling toward mitophagy and its physiological significance. *Experimental Cell Research*.

[107] Ginet V., Puyal J., Clarke P. G. H., Truttmann A. C. (2009). Enhancement of autophagic flux after neonatal cerebral hypoxia-ischemia and its region-specific relationship to apoptotic mechanisms. *The American Journal of Pathology*.

[108] Koike M., Shibata M., Tadakoshi M. (2008). Inhibition of autophagy prevents hippocampal pyramidal neuron death after hypoxic-ischemic injury. *The American Journal of Pathology*.

[109] Papadakis M., Hadley G., Xilouri M. (2013). Tsc1 (hamartin) confers neuroprotection against ischemia by inducing autophagy. *Nature Medicine*.

[110] Puyal J., Vaslin A., Mottier V., Clarke P. G. H. (2009). Postischemic treatment of neonatal cerebral ischemia should target autophagy. *Annals of Neurology*.

[111] Wei K., Wang P., Miao C. Y. (2012). A double-edged sword with therapeutic potential: an updated role of autophagy in ischemic cerebral injury. *CNS Neuroscience & Therapeutics*.

[112] Chen Y., Azad M. B., Gibson S. B. (2009). Superoxide is the major reactive oxygen species regulating autophagy. *Cell Death & Differentiation*.

[113] Huang J., Canadien V., Lam G. Y. (2009). Activation of antibacterial autophagy by NADPH oxidases. *Proceedings of the National Academy of Sciences of the United States of America*.

[114] Frank M., Duvezin-Caubet S., Koob S. (2012). Mitophagy is triggered by mild oxidative stress in a mitochondrial fission dependent manner. *Biochimica et Biophysica Acta (BBA) - Molecular Cell Research*.

[115] Mahalingaiah P. K. S., Singh K. P. (2014). Chronic oxidative stress increases growth and tumorigenic potential of mcf-7 breast cancer cells. *PLoS One*.

[116] Byun Y. J., Kim S. K., Kim Y. M., Chae G. T., Jeong S. W., Lee S. B. (2009). Hydrogen peroxide induces autophagic cell death in C6 glioma cells via BNIP3-mediated suppression of the mTOR pathway. *Neuroscience Letters*.

[117] Zhang L., Wang H., Xu J., Zhu J., Ding K. (2014). Inhibition of cathepsin S induces autophagy and apoptosis in human glioblastoma cell lines through ROS-mediated PI3K/AKT/mTOR/p70S6K and JNK signaling pathways. *Toxicology Letters*.

[118] Marin J. J. G., Lozano E., Perez M. J. (2016). Lack of mitochondrial DNA impairs chemical hypoxia-induced autophagy in liver tumor cells through ROS-AMPK-ULK1 signaling dysregulation independently of HIF-1α. *Free Radical Biology & Medicine*.

[119] Wen X., Wu J., Wang F., Liu B., Huang C., Wei Y. (2013). Deconvoluting the role of reactive oxygen species and autophagy in human diseases. *Free Radical Biology & Medicine*.

[120] Jin S. (2006). Autophagy, mitochondrial quality control, and oncogenesis. *Autophagy*.

[121] Wu J. J., Quijano C., Chen E. (2009). Mitochondrial dysfunction and oxidative stress mediate the physiological impairment induced by the disruption of autophagy. *Aging*.

[122] Rubinsztein D. C., Codogno P., Levine B. (2012). Autophagy modulation as a potential therapeutic target for diverse diseases. *Nature Reviews Drug Discovery*.

[123] Taguchi K., Motohashi H., Yamamoto M. (2011). Molecular mechanisms of the Keap1–Nrf2 pathway in stress response and cancer evolution. *Genes to Cells*.

[124] Villeneuve N. F., Lau A., Zhang D. D. (2010). Regulation of the Nrf2–Keap1 antioxidant response by the ubiquitin proteasome system: an insight into cullin-ring ubiquitin ligases. *Antioxidants & Redox Signaling*.

[125] Bjørkøy G., Lamark T., Brech A. (2005). p62/SQSTM1 forms protein aggregates degraded by autophagy and has a protective effect on huntingtin-induced cell death. *The Journal of Cell Biology*.

[126] Ichimura Y., Waguri S., Sou Y.-s. (2013). Phosphorylation of p62 activates the Keap1-Nrf2 pathway during selective autophagy. *Molecular Cell*.

[127] Taguchi K., Fujikawa N., Komatsu M. (2012). Keap1 degradation by autophagy for the maintenance of redox homeostasis. *Proceedings of the National Academy of Sciences of the United States of America*.

[128] da-Silva W. S., Gómez-Puyou A., de Gómez-Puyou M. T. (2004). Mitochondrial bound hexokinase activity as a preventive antioxidant defense: steady-state ADP formation as a regulatory mechanism of membrane potential and reactive oxygen species generation in mitochondria. *Journal of Biological Chemistry*.

[129] Roberts D. J., Tan-Sah V. P., Ding E. Y., Smith J. M., Miyamoto S. (2014). Hexokinase-II positively regulates glucose starvation-induced autophagy through TORC1 inhibition. *Molecular Cell*.

[130] Li L., Tan J., Miao Y., Lei P., Zhang Q. (2015). ROS and autophagy: interactions and molecular regulatory mechanisms. *Cellular and Molecular Neurobiology*.

[131] Scherz-Shouval R., Elazar Z. (2007). ROS, mitochondria and the regulation of autophagy. *Trends in Cell Biology*.

[132] Yakes F. M., Van Houten B. (1997). Mitochondrial DNA damage is more extensive and persists longer than nuclear DNA damage in human cells following oxidative stress. *Proceedings of the National Academy of Sciences of the United States of America*.

[133] Joselin A. P., Hewitt S. J., Callaghan S. M. (2012). ROS-dependent regulation of Parkin and DJ-1 localization during oxidative stress in neurons. *Human Molecular Genetics*.

[134] Yang S., Xia C., Li S., Du L., Zhang L., Zhou R. (2014). Defective mitophagy driven by dysregulation of rheb and KIF5B contributes to mitochondrial reactive oxygen species (ROS)-induced nod-like receptor 3 (NLRP3) dependent proinflammatory response and aggravates lipotoxicity. *Redox Biology*.

[135] Flynn J. M., Melov S. (2013). SOD2 in mitochondrial dysfunction and neurodegeneration. *Free Radical Biology & Medicine*.

[136] Kirkland R. A., Adibhatla R. M., Hatcher J. F., Franklin J. L. (2002). Loss of cardiolipin and mitochondria during programmed neuronal death: evidence of a role for lipid peroxidation and autophagy. *Neuroscience*.

[137] Cherra S. J., Chu C. T. (2008). Autophagy in neuroprotection and neurodegeneration: a question of balance. *Future Neurology*.

[138] Carloni S., Buonocore G., Balduini W. (2008). Protective role of autophagy in neonatal hypoxia–ischemia induced brain injury. *Neurobiology of Disease*.

[139] Redmann M., Darley-Usmar V., Zhang J. (2016). The role of autophagy, mitophagy and lysosomal functions in modulating bioenergetics and survival in the context of redox and proteotoxic damage: implications for neurodegenerative diseases. *Aging and Disease*.

[140] Kahles T., Brandes R. P. (2013). Which NADPH oxidase isoform is relevant for ischemic stroke? The case for nox 2. *Antioxidants & Redox Signaling*.

[141] Albers G. W., Caplan L. R., Easton J. D. (2002). Transient ischemic attack — proposal for a new definition. *The New England Journal of Medicine*.

[142] Manzanero S., Santro T., Arumugam T. V. (2013). Neuronal oxidative stress in acute ischemic stroke: sources and contribution to cell injury. *Neurochemistry International*.

[143] Adhami F., Schloemer A., Kuan C. Y. (2007). The roles of autophagy in cerebral ischemia. *Autophagy*.

[144] Zhao Y., Huang G., Chen S., Gou Y., Dong Z., Zhang X. (2016). Folic acid deficiency increases brain cell injury via autophagy enhancement after focal cerebral ischemia. *The Journal of Nutritional Biochemistry*.

[145] Wang W., Sun Y., Dai M., Tang Y., Sun Q., Bian L. (2013). The regulation effect of oxidative stress on autophagy after cerebral ischemia-reperfusion injury. *Chinese Journal of Minimally Invasive Neurosurgery*.

[146] Kubota C., Torii S., Hou N. (2010). Constitutive reactive oxygen species generation from autophagosome/lysosome in neuronal oxidative toxicity. *Journal of Biological Chemistry*.

[147] Dai S. H., Chen T., Li X. (2017). Sirt3 confers protection against neuronal ischemia by inducing autophagy: involvement of the AMPK-mTOR pathway. *Free Radical Biology & Medicine*.

[148] Cheng A., Yang Y., Zhou Y. (2016). Mitochondrial SIRT3 mediates adaptive responses of neurons to exercise and metabolic and excitatory challenges. *Cell Metabolism*.

[149] Shao J., Yang X., Liu T., Zhang T., Xie Q. R., Xia W. (2016). Autophagy induction by SIRT6 is involved in oxidative stress-induced neuronal damage. *Protein & Cell*.

[150] Yuan Y., Zhang X., Zheng Y., Chen Z. (2015). Regulation of mitophagy in ischemic brain injury. *Neuroscience Bulletin*.

[151] Von Bernhardi R., Eugenín J. (2012). Alzheimer’s disease: redox dysregulation as a common denominator for diverse pathogenic mechanisms. *Antioxidants & Redox Signaling*.

[152] Moreira P. I., Santos M. S., Oliveira C. R. (2007). Alzheimer’s disease: a lesson from mitochondrial dysfunction. *Antioxidants & Redox Signaling*.

[153] de la Monte S. M., Neely T. R., Cannon J., Wands J. R. (2000). Oxidative stress and hypoxia-like injury cause Alzheimer-type molecular abnormalities in central nervous system neurons. *Cellular and Molecular Life Sciences*.

[154] Giordano S., Darley-Usmar V., Zhang J. (2014). Autophagy as an essential cellular antioxidant pathway in neurodegenerative disease. *Redox Biology*.

[155] Underwood B. R., Imarisio S., Fleming A. (2010). Antioxidants can inhibit basal autophagy and enhance neurodegeneration in models of polyglutamine disease. *Human Molecular Genetics*.

[156] Dodson M., Darley-Usmar V., Zhang J. (2013). Cellular metabolic and autophagic pathways: traffic control by redox signaling. *Free Radical Biology & Medicine*.

[157] Barbero-Camps E., Fernández A., Martínez L., Fernández-Checa J. C., Colell A. (2013). APP/PS1 mice overexpressing SREBP-2 exhibit combined Aβ accumulation and tau pathology underlying Alzheimer’s disease. *Human Molecular Genetics*.

[158] Gobe G., Crane D. (2010). Mitochondria, reactive oxygen species and cadmium toxicity in the kidney. *Toxicology Letters*.

[159] Dagda R. K., Cherra S. J., Kulich S. M., Tandon A., Park D., Chu C. T. (2009). Loss of PINK1 function promotes mitophagy through effects on oxidative stress and mitochondrial fission. *Journal of Biological Chemistry*.

[160] Thomas K. J., McCoy M. K., Blackinton J. (2011). DJ-1 acts in parallel to the PINK1/parkin pathway to control mitochondrial function and autophagy. *Human Molecular Genetics*.

[161] Miyazaki S., Yanagida T., Nunome K. (2008). DJ-1-binding compounds prevent oxidative stress-induced cell death and movement defect in Parkinson’s disease model rats. *Journal of Neurochemistry*.

[162] Wen Y. D., Sheng R., Zhang L. S. (2008). Neuronal injury in rat model of permanent focal cerebral ischemia is associated with activation of autophagic and lysosomal pathways. *Autophagy*.

[163] Yang Y., Gao K., Hu Z. (2015). Autophagy upregulation and apoptosis downregulation in DAHP and triptolide treated cerebral ischemia. *Mediators of Inflammation*.

[164] Manczak M., Anekonda T. S., Henson E., Park B. S., Quinn J., Reddy P. H. (2006). Mitochondria are a direct site of Aβ accumulation in Alzheimer’s disease neurons: implications for free radical generation and oxidative damage in disease progression. *Human Molecular Genetics*.

[165] Manczak M., Mao P., Calkins M. J. (2010). Mitochondria-targeted antioxidants protect against amyloid-β toxicity in Alzheimer’s disease neurons. *Journal of Alzheimer's Disease*.

